# Enhanced recovery mitigates sodium-glucose cotransporter-2 inhibitors associated mobility decline in valve surgery patients

**DOI:** 10.3389/fcvm.2026.1746050

**Published:** 2026-02-24

**Authors:** Chengyao Xu, Minlai Chen, Enkang Lu, Xuezhou Zhang, Hao You, Xia Zhao, Yong Sun, Xin Xu

**Affiliations:** 1 School of Sports and Health, Shanghai University of Sport, Shanghai, China; 2 Department of Cardiovascular Surgery, Zhongshan Hospital Affiliated to Xiamen University, Xiamen, China

**Keywords:** enhanced recovery after surgery, mobility, perioperative rehabilitation, SGLT2 inhibitors, valvular heart disease

## Abstract

**Background:**

Sodium-glucose cotransporter-2 inhibitors (SGLT2i) are integral to quadruple therapy for perioperative heart failure management in valvular heart disease (VHD) patients. Despite cardioprotective benefits, SGLT2i use correlates with acute sarcopenia and mobility impairment. This study evaluated whether Enhanced Recovery After Surgery (ERAS) protocols mitigate SGLT2i-associated mobility limitations.

**Methods:**

In a single-center, retrospective cohort study [conducted from (Start Date) to (End Date)], 48 VHD patients undergoing valve surgery under an Enhanced Recovery After Surgery (ERAS) protocol were analyzed (Quadruple therapy [QT] with SGLT2i, *n* = 34; Triple therapy [TT] without SGLT2i, *n* = 14). Primary outcomes assessed mobility via Activities of Daily Living (ADL, Barthel Index), stress ulcer risk (Braden scale), and fall risk (Modified Thomas scale) at multiple perioperative timepoints. Secondary outcomes included ERAS metrics (ICU duration, mobilization times), cardiac function, and laboratory parameters.

**Results:**

The QT group experienced significantly longer ICU stays than the TT group (median 49 vs. 43 h, *p* = 0.003), indicating greater initial morbidity and resource utilization. The time to first off-bed mobilization was also delayed in the QT group (67 vs. 53 h, *p* = 0.042), a 14-hour delay that heightens the risk of muscle atrophy and pulmonary complications. Post-anesthesia recovery: QT showed lower ADL scores (10 vs. 15, *p* = 0.007) and higher stress ulcer risk (Braden 13 vs. 16, *p* = 0.025). ICU discharge: QT maintained higher stress ulcer risk (Braden 16 vs. 17, *p* = 0.006). Secondary care: QT demonstrated superior ADL recovery (95 vs. 85, *p* = 0.017) and lower stress ulcer risk (Braden 22 vs. 21, *p* = 0.043). No intergroup differences in cardiac function or laboratory parameters (*p* > 0.05). Multivariate analysis identified ICU duration, mechanical ventilation time, platelets, albumin, and NT-proBNP as significant mobility correlates (|r| = 0.368–0.625, *p* < 0.05).

**Conclusion:**

SGLT2i use is associated with transient perioperative mobility impairment in VHD patients, evidenced by longer ICU stays and delayed mobilization. However, a structured ERAS protocol effectively mitigates these limitations, facilitating superior functional recovery by discharge. These findings underscore the necessity of adapting or closely monitoring ERAS protocols, particularly for patients on SGLT2i, to mitigate early mobility deficits. This supports tailoring ERAS pathways, particularly early mobilization, for this patient population.

## 1 Introduction

Valvular heart disease (VHD) remains a global health burden, with rheumatic heart disease affecting 41 million people worldwide. Calcific aortic stenosis and degenerative mitral valve disease affect approximately 9 million and 24 million individuals, respectively (1). Current perioperative heart failure guidelines recommend quadruple therapy, combining ACEI/ARB/ARNI, beta-blockers, aldosterone antagonists, and increasingly SGLT2 inhibitors (SGLT2i) (2). Despite established cardioprotective mechanisms, the mechanistic profile of SGLT2i necessitates nuanced evaluation. Preclinical evidence indicates potential metabolic trade-offs, such as the activation of muscle-specific ubiquitin ligases, which may promote proteolysis and reduce lean mass. SGLT2i activate muscle-specific ubiquitin ligases (MAFbx/atrogin-1, MuRF1), triggering proteolytic pathways that may reduce lean mass despite preserved cardiac function (3–5).

SGLT2 inhibitors demonstrate conflicting musculoskeletal consequences: clinical reports note sarcopenia development (e.g., reduced limb circumference, grip strength <18 kg in females) within 30 days of dapagliflozin initiation in type II diabetes (6). Yet their impact on mobility remains contested. Meta-analyses indicate improved VO_2_max, ventilatory anaerobic threshold, and exercise endurance in diabetes-heart failure cohorts (7), while animal models suggest acute muscle alterations reflect adaptive metabolic shifts—enhanced fat oxidation, mitochondrial biogenesis, and amino acid mobilization for gluconeogenesis—rather than pathological atrophy (8, 9). This paradox necessitates reconciling SGLT2i's survival benefits with potential mobility impairment, which we operationalized using the Barthel Index for Activities of Daily Living. Crucially, mobility changes in valvular heart disease (VHD) patients receiving SGLT2i are unstudied. Findings from heart failure or diabetic cohorts may not be directly generalizable to VHD patients due to differences in pathophysiology, such as the impact of pressure or volume overload on musculoskeletal function. Therefore, investigating, despite perioperative functional capacity being inversely correlated with 1-year postoperative mortality. Diminished mobility consistently predicts adverse surgical outcomes, underscoring its role as a cardinal perioperative evaluation metric for VHD populations.

In this context, Enhanced Recovery after Surgery (ERAS) protocols are pertinent for optimizing mobility in patients undergoing VHD surgery (10). ERAS protocols mandate comprehensive preoperative assessment with exercise tolerance testing to guide individualized pre-rehabilitation targeting functional reserve augmentation. Core components include patient education and early postoperative mobilization through structured rehabilitation. Early ambulation critically accelerates multisystem recovery (respiratory, musculoskeletal, digestive), reducing pulmonary complications, stress ulceration, and venous thromboembolism risks. In VHD patients, perioperative interventions—preoperative 6-minute walk tests, respiratory training, daily walking (>30 min), and postoperative low-intensity resistance training with balance exercises—significantly improve muscle strength, mobility, and mental health outcomes. These benefits directly correlate with enhanced postoperative quality of life (11–14). We hypothesized that within a standardized ERAS pathway, the use of SGLT2 inhibitors would not lead to inferior perioperative mobility outcomes compared to triple therapy in patients undergoing valve surgery. This study aimed to test this hypothesis by comparing recovery trajectories by comparing recovery trajectories between patients on quadruple (SGLT2i-inclusive) and triple therapy after valve surgery.

## 2 Materials and methods

### 2.1 Study design and population

This single-center, retrospective cohort study conducted at Zhongshan Hospital, Xiamen University, which obtained ethics approval (2024–113) and adhered to the Declaration of Helsinki, enrolled patients with VHD undergoing elective valve surgery within ERAS protocol.

The selected cases obtained verbal informed consent during the follow-up calls. Data were extracted from the institutional Hospital Information System-electronic medical record (HIS-EMR). Inclusion criteria were age 18–75 years, New York Heart Association (NYHA) class I–III, absence of significant extra-cardiac organ dysfunction or hepatorenal disease history, and no conditions like pulmonary edema that could interfere with the intervention. Exclusion criteria included NYHA class IV, major organ dysfunction, age ≥75 years, or significant pulmonary/limb edema. After screening, 48 eligible patients were included in this retrospective analysis. The sample size was determined by the available cohort during the study period. The assignment to the quadruple therapy (QT, *n* = 34) or triple therapy (TT, *n* = 14) group was determined by the treating physician based on prevailing heart failure guidelines and individual patient clinical status at admission. Patients in the QT group received a SGLT2 inhibitor (e.g., dapagliflozin 10 mg/day or empagliflozin 10 mg/day) as part of their regimen. Medication administration followed institutional protocols, typically continuing until the day before surgery, continue to take the medication when regaining the ability to swallow after anesthesia recovery.

It is important to note that this was not a randomized allocation. Consequently, significant baseline differences were observed, such as lower diastolic and systolic blood pressure in the QT group, which may reflect differences in underlying cardiac function and represent potential confounding factors for postoperative recovery outcomes. All patients were managed according to the institutional ERAS path-way, and baseline demographics, clinical characteristics, VHD classifications, and surgical types were recorded (Supplementary Table S1). The primary outcome was mobility, assessed using the Barthel Index for Activities of Daily Living (ADL). Secondary outcomes included the Braden scale for stress ulcer risk and the Modified Thomas scale for fall risk. These outcomes were measured at five timepoints: hospital admission, post-anesthesia care unit (PACU) discharge, ICU discharge, transition to secondary care, and final hospital discharge.

### 2.2 ERACS protocol components and assessment

The study implemented a three-phase ERAS protocol for elective valve surgery (15, 16). The protocol included preoperative respiratory and walking exercises, intraoperative goal-directed fluid therapy and myocardial protection, and postoperative early mobilization and analgesia. Patient mobility and recovery metrics were assessed perioperatively using standardized scales.(Barthel Index for activities of daily living (17), the Braden scale for stress ulcer risk (18), and the Modified Thomas Scale for fall risk (19). (Supplementary Table S2), and outcomes were evaluated based on operative times, ICU stay, mobilization milestones, and cardiac function.

Preoperatively phase began upon admission and continued until surgery, encompassing respiratory training (diaphragmatic breathing: slow 3–5 s inhalation with abdominal expansion, 1-second breath-hold, controlled 3–5 s exhalation with abdominal retraction, followed by a 1-second pause, performed 20 times daily) and walking exercises (15–30 min, 1–2 times daily). Prophylactic antibiotics, patient education, and psychological support were also provided to mitigate surgical risks.

Intraoperatively, goal-directed fluid therapy, guided by transesophageal echocardiography, was employed for stroke volume optimization. Key techniques included cardiopulmonary bypass (CPB), antegrade cold-blooded cardioplegia, modified ultrafiltration, retrograde warm blood perfusion, and near-infrared spectroscopy monitoring to ensure cerebral oxygen saturation, plasma colloid osmotic pressure, and myocardial protection.

Postoperatively, patients received analgesia, anti-inflammatory agents, and early mobilization protocols, including bed-based limb exercises, early catheter removal, and supervised ambulation. Criteria for Intensive Care Unit (ICU) discharge included hemodynamic stability (urine output >1 mL/kg/h, independence from vasoactive drugs) and oxygenation adequacy (SpO_2_ > 90%). Ward-based rehabilitation advanced from seated exercises to independent walking (150–200 meters). Patients were discharged upon achieving stable cardiac function, independence in daily activities, and echocardiographic confirmation of valve integrity. This comprehensive protocol demonstrably reduced postoperative complications and expedited functional recovery.

### 2.3 Statistical analysis

Statistical analyses were executed utilizing IBM SPSS Statistics 25.0 (SPSS Inc., Chicago, IL, USA). Before conducting hypothesis tests, data distribution was systematically assessed via the Shapiro–Wilk test for normality and Levene's test for homogeneity of variances. For comparisons between groups, parametric datasets were analyzed using an independent samples *t*-test with Welch's correction. Non-normally distributed data, however, were subjected to the Mann–Whitney *U*-test, with exact significance computation. Within-group comparisons involving repeated measures were conducted using the Friedman test, followed by Bonferroni-adjusted *post-hoc* analyses. The level of statistical significance was predefined at α = 0.05 (two-tailed). Given the observed baseline differences (e.g., blood pressure), their potential influence was considered in the interpretation of correlations. Analyses were performed using complete cases only; no imputation was conducted for missing data. The strength of these correlations was interpreted based on the following established benchmarks: ∣r∣ > 0.70 (very strong), 0.50–0.69 (strong), 0.30–0.49 (moderate), 0.10–0.29 (weak), and <0.10 (negligible).

## 3 Results

Analysis of mobility trajectories revealed a dynamic recovery pattern. The study enrolled 48 patients, with comparable baseline characteristics between the QT group (*n* = 34) and TT group (*n* = 14). Median age was similar across both groups (median: 66 vs. 64 years; *p* = 0.81). Gender distribution demonstrated a male predominance in the QT group (20 males, 58.82%; 14 females, 41.18%), whereas the TT group showed a balanced sex ratio (6 males, 42.86%; 8 females, 57.14%). Compared with the TT group, the diastolic and systolic blood pressures in the QT group were significantly lower. (DBP median: 165.50 vs. 189.50 mmHg, SBP median: 92.50 vs. 116.50 mmHg, *p* < 0.01). Baseline characteristics showed significantly higher diastolic and systolic blood pressures in the QT group compared to the TT group (Supplementary Table S3).

### 3.1 ERAS protocol components and mobility assessment of VHD patients

As shown in Table 1. When benchmarked against ERAS protocol targets, the QT group exhibited a statistically significant difference in duration of ICU duration compared to the TT group (median: 49 vs. 43 h; *p* = 0.003). Similarly, the time to first off-bed mobilization was longer in the QT group (median: 67 vs. 53 h; *p* = 0.042). No significant intergroup differences were observed in other intraoperative or postoperative ERAS elements.

**Table 1:** ERAS protocol components between quadruple therapy group and triple therapy group.

ERAS report elements	QT (*n* = 34)	TT (*n* = 14)		
	M (Q1, Q3)	M (Q1, Q3)	*p* Value^a^	*Z* value
Duration of operation (h)	7.00 (6.50,8.00)	6.50 (5.75,7.50)	0.648	−0.457
Duration of mechanical ventilation (h)	14.00 (5.00,18.00)	6.00 (4.00,14.00)	0.170	−1.373
Duration of ICU (h)	49.00 (43.00,67.00)**	43.00 (26.00,55.00)	0.003	−3.008
Time of first in-bed mobilization after surgery (h)	17.00 (5.00,41.00)	27.00 (10.00,89.50)	0.273	−1.137
Time of first off-bed mobilization after surgery (h)	67.00 (45.00,114.00)*	53.00 (42.75,89.50)	0.042	−2.026

a Non-parametric analyses were performed using the Mann–Whitney *U*-test with Bonferroni correction for multiple comparison.

* *p* < 0.05.

** *p* < 0.01.Comparison of key ERAS protocol metrics between the Quadruple Therapy (QT) and Triple Therapy (TT) groups. Data presented as Median (Q1, Q3). The QT group had a significantly longer ICU duration and delayed time to first off-bed mobilization (* *p* < 0.05, ** *p* < 0.01, Mann–Whitney *U*-test).

**Figure 1:**
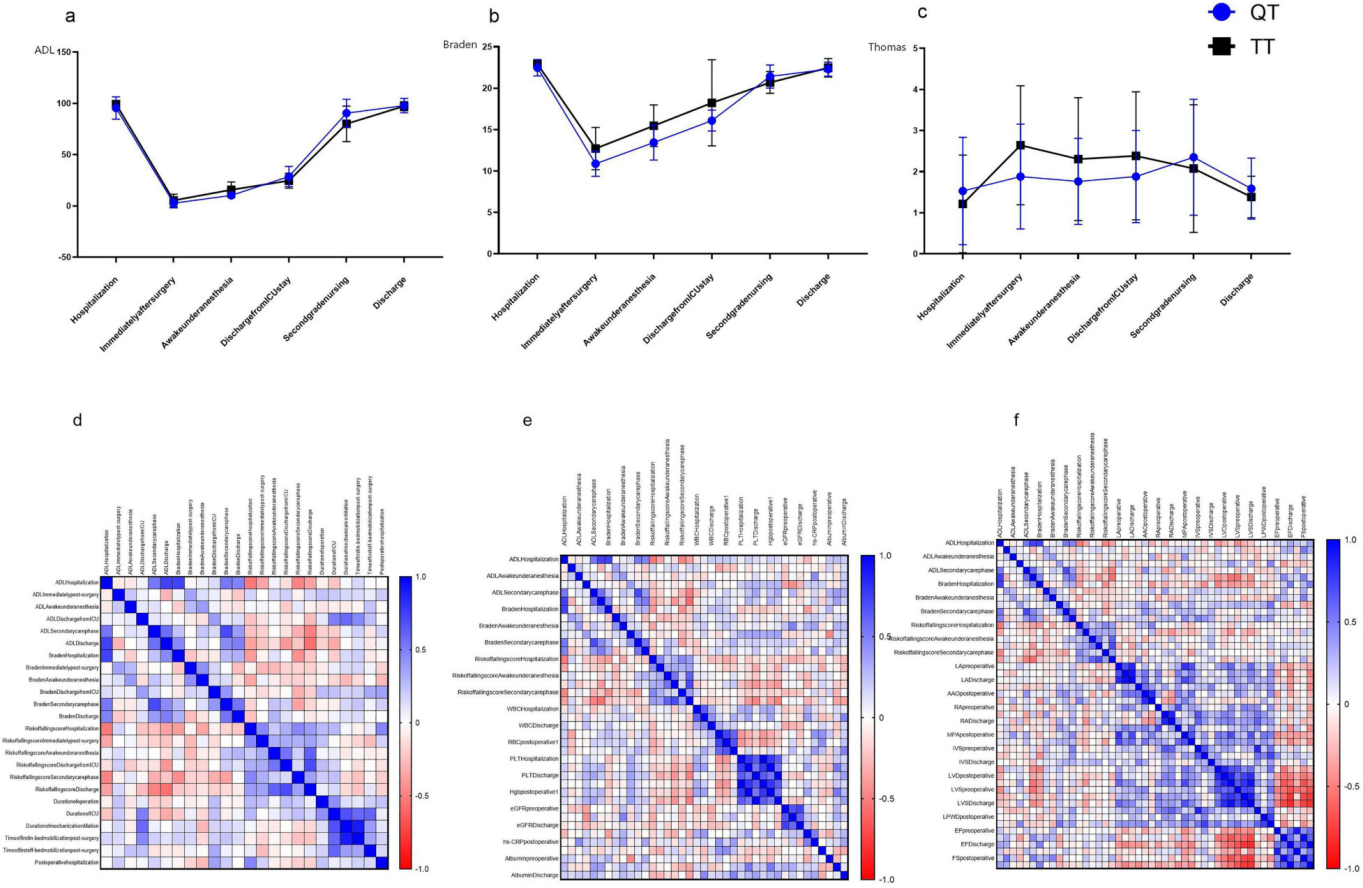
Perioperative mobility trajectories and their correlates. **(a–c)** Temporal changes in Activities of Daily Living (ADL, **a**), stress ulcer risk (Braden scale, **b**), and fall risk (Modified Thomas scale, **c**) for the Quadruple Therapy (QT) and Triple Therapy (TT) groups. Note the initial deficit in QT patients during anesthesia recovery, followed by a crossover and superior performance in ADL during secondary care. **(d–f)** Correlation heatmaps of mobility assessments with ERAS components **(d)**, laboratory parameters **(e)**, and cardiac function **(f)**.

Both groups exhibited significant mobility improvements following ERAS-guided early mobilization interventions (Figures 1a–c). Activities of Daily Living (ADL) scores reached their nadir immediately post-surgery but progressively recovered, returning to preoperative levels by discharge (QT group: *χ*^2^ = 160.11, *p* < 0.01; TT group: *χ*^2^ = 61.99, *p* < 0.01). Similarly, Braden scales—indicating peak stress ulcer risk—were lowest postoperatively, with risk markedly reduced through mobilization (QT group: *χ*^2^ = 158.19, *p* < 0.01; TT group: *χ*^2^ = 58.80, *p* < 0.01). Modified Thomas fall risk assessment scale shows that there were significant changes in the intra-group comparison of risk of falling in both groups of patients. (QT group: *χ*^2^ = 14.245, *p* < 0.05; TT group: *χ*^2^ = 58.80, *p* < 0.01) While triple therapy patients demonstrated declining fall risk post-intervention, the QT group paradoxically showed escalating fall risk during initial ERAS implementation, peaking at secondary care transition before decreasing at discharge.

Intergroup analysis demonstrated differential ADL recovery trajectories between groups. During the post-anesthesia recovery phase, the QT group exhibited notably lower ADL scores compared to the TT group (median: 10 vs. 15; *p* = 0.007). Conversely, this pattern reversed during secondary care, with the QT group achieving significantly higher ADL functionality (median: 95 vs. 85; *p* = 0.017), suggesting accelerated late-phase rehabilitation efficacy in this group. Braden scale assessments revealed distinct stress ulcer risk profiles between groups. The QT group demonstrated significantly lower Braden scales (indicating higher ulcer risk) than the TT group during post-anesthesia recovery (median: 13 vs. 16; *p* = 0.025) and at discharge from ICU (median: 16 vs. 17; *p* = 0.006). The risk of stress ulcers was higher in the QT group. However, this trend reversed during the secondary care phase, (median: 22 vs. 21; *p* = 0.043) where the QT group demonstrated a significantly lower risk of stress ulcers. Inter-group analysis demonstrated no statistically significant intergroup differences in Modified Thomas fall risk assessment scale across all five assessment time points (all *p* > 0.05). The results of functional mobility assessement intra and intergroup comparison presented in Supplementary Table S4.

### 3.2 The influence of early postoperative activities on cardiac function and laboratory test indicators

No significant differences were observed in cardiac structure/function or laboratory parameters between the QT and TT groups during postoperative and discharge assessments (Mann–Whitney *U*-test, all *p* > 0.05) (Supplementary Table S5). The comparable parameters included left atrial diameter, ascending aortic diameter, right atrial diameter, main pulmonary artery diameter, interventricular septal thickness, left ventricular end-diastolic diameter, ejection fraction, fractional shortening, white blood cell count, red blood cell count, hemoglobin, platelets, epidermal growth factor receptor (eGFR), and serum albumin levels.

### 3.3 Correlation analysis

To identify determinants of early mobilization efficacy, a multivariate correlation analysis was performed within the QT group. This analysis assessed the relationships between mobility indicators, ERAS protocol compliance parameters, cardiac functional indices, and laboratory biomarkers in medication-adherent patients.

Analysis of perioperative parameters revealed significant correlations with functional outcomes (Figure 1d). Mechanical ventilation duration positively correlated with ADL at ICU discharge (*r* = 0.581, *p* < 0.001) but negatively correlated with ADL at admission (*r* = −0.355, *p* = 0.039) and during secondary care (*r* = −0.382, *p* = 0.026). Similarly, ICU stay duration showed a positive correlation with ADL at ICU discharge (*r* = 0.533, *p* = 0.001) and a negative correlation with the Braden scale at ICU discharge (*r* = −0.412, *p* = 0.015). Earlier in-bed mobilization was associated with better functional outcomes at ICU discharge (*r* = 0.414, *p* = 0.017). A longer postoperative hospitalization was associated with a higher stress ulcer risk (Braden scale at ICU discharge: *r* = 0.405, *p* = 0.017) and an elevated fall risk (Modified Thomas scale).

Analysis of laboratory parameters revealed significant correlations with functional outcomes (Figure 1e). Platelet count (PLT) at discharge demonstrated protective effects, showing strong negative correlations with fall risk (TRAFS) at awakening (*r* = −0.370, *p* = 0.034), ICU discharge (*r* = −0.520, *p* = 0.002), and final discharge (*r* = −0.589, *p* < 0.001). Conversely, elevated NT-proBNP at discharge was associated with impaired functional recovery, correlating negatively with ADL during secondary care (*r* = −0.480, *p* = 0.005) and the Braden scale at awakening (*r* = −0.585, *p* < 0.001). Preoperative albumin levels appeared beneficial, showing positive correlations with ADL in secondary care (*r* = 0.368, *p* = 0.032) and the Braden scale at admission (*r* = 0.442, *p* = 0.009). A paradoxical association was observed for postoperative eGFR, which correlated negatively with ADL at ICU discharge (*r* = −0.395, *p* = 0.023) but positively with the Braden scale at awakening (*r* = 0.547, *p* = 0.001). Additionally, admission RBC count negatively correlated with postoperative ADL (*r* = −0.398, *p* = 0.020), while postoperative PLT positively correlated with ADL at ICU discharge (*r* = 0.393, *p* = 0.022).

Cardiovascular Parameter Correlations with Functional Outcomes (Figure 1f): Aortic Root (AAO): Preoperative diameter positively correlated with fall risk (TRAFS) at ICU discharge (*r* = 0.406, *p* = 0.021) and final discharge (*r* = 0.408, *p* = 0.020). Main Pulmonary Artery (MPA): Preoperative diameter positively correlated with stress ulcer risk (Braden scale) at ICU discharge (*r* = 0.526, *p* = 0.002). Interventricular Septum (IVS): Preoperative thickness showed the strongest positive correlation with Braden scale at discharge (*r* = 0.625, *p* < 0.001). Left Ventricular Diameter (LVD): Preoperative and postoperative diameters were negatively correlated with ADL scores at discharge (preoperative: *r* = −0.404, *p* = 0.022; postoperative: *r* = −0.522, *p* = 0.006). Ejection Fraction (EF) & Fractional Shortening (FS): Postoperative EF and FS were positively correlated with multiple functional recovery metrics, including ADL at discharge (EF: *r* = 0.462; FS: *r* = 0.484) and Braden scale at awakening (FS: *r* = 0.609). Correlation data can be found in Supplementary Tables S6–S8.

## 4 Discussion

### 4.1 The impact of SGLT-2i on the perioperative rehabilitation status, activity ability and cardiac function of patients with VHD

Comparative analysis shows that patients receiving quadruple therapy (QT) containing SGLT-2i have significant differences in enhanced recovery after surgery (ERAS) reporting elements compared with those receiving triple therapy (TT), manifested as prolonged ICU stay and delayed first ambulation time, thereby increasing the risk of postoperative pulmonary and thromboembolic complications and the consumption of medical resources (20, 21). Early getting out of bed and moving around is the cornerstone of postoperative rehabilitation after cardiac surgery. However, the impaired functional recovery of patients in the QT group indicates that their neuromuscular rehabilitation is inhibited. As a component of quadruple therapy, our finding of delayed early mobilization in the QT group suggests that SGLT-2i may potentially exacerbate motor dysfunction, possibly through mechanisms promoting catabolism and potentially having a muscle-reducing effect (22–24).

The perioperative activities of daily living (ADL) score was significantly correlated with laboratory indicators of inflammation, nutrition and cardiac function, revealing the multi-level physiological mechanisms of functional recovery. The ADL score is positively correlated with hemoglobin, platelet count and serum albumin, emphasizing the role of oxygen delivery, coagulation capacity and nutritional status in functional recovery (25). Organizational Doppler analysis linked impaired functional activity to left ventricular dilation and systolic dysfunction, while maintaining good cardiac output was associated with better activity. Although SGLT-2i can improve cardiac function, the potential negative impact on muscle mass observed cannot be ignored.

However, the subsequent functional recovery trajectory analysis of the QT group revealed an important clinical phenomenon: despite the challenges in the early stage of anesthesia recovery, patients in this group demonstrated accelerated functional benefits during postoperative hospitalization with the support of the structured ERAS protocol. This bidirectional recovery model emphasizes two fundamental surgical principles: First, there is a dynamic interaction between drug-induced neural regulation and the intensity of functional rehabilitation; Second, there is a negative correlation between activity limitations and the survival ability of an organization. The reduction in the risk of stress ulcers in the QT group also indicates from the side that rehabilitation exercise has a positive effect on reducing the side effects of SGLT-2i drugs.

### 4.2 The comprehensive benefits of the ERAS protocol for patients with valvular heart disease using SGLT-2i

The subsequent functional recovery of the QT group highlighted the crucial role of the ERAS protocol. The standardized and proactive activity program inherent in the ERAS protocol may serve as an effective anabolic countermeasure. By enforcing early and progressive physical activity, ERAS is likely to promote mitochondrial biosynthesis and metabolic flexibility, thereby offsetting the catabolic stress jointly induced by surgery and SGLT-2i. This is consistent with the research finding that “earlier bedside activities are associated with better functional outcomes when transferred out of the ICU” (26–30).

Activity intervention, as a core component of rehabilitation physiology, especially a crucial part of the ERAS protocol, can effectively counteract the catabolic state that SGLT-2i may induce. As a key stimulus, exercise activates mitochondrial biogenesis through signaling pathways such as AMPK/PGC-1α and optimizes the regulation of mitochondrial dynamics. The metabolic state similar to starvation induced by SGLT-2i treatment (manifested as hypoglycemia and high ketone bodies) may exacerbate the tendency towards catabolism. However, the ERAS strategy, which combines early postoperative activity with exercise intervention, enhances the utilization efficiency of glucose and fatty acids by muscles and reduces the surgical stress response (such as lowering cortisol levels), thereby synergistically improving energy metabolism balance and optimizing patient prognosis (31–33).

These findings collectively advocate that for patients receiving metabolic regulators (such as SGLT-2i), a stratified rehabilitation model should be adopted, emphasizing targeted activities in the early stage to counteract drug-induced sarcopenia, while maintaining vigilance against delayed functional compensation during the rehabilitation phase. Ultimately, the ERAS strategy that combines early postoperative intervention with exercise provides a crucial biological basis for the accelerated recovery of patients undergoing cardiovascular surgery by enhancing mitochondrial functional volume and metabolic adaptability.

### 4.3 Blood pressure as an element for risk assessment in enhanced recovery

At baseline, the QT group exhibited significantly lower diastolic and systolic blood pressure than the TT group. In heart failure, such hypotension often reflects impaired cardiac output and inadequate tissue perfusion, indicating severe cardiac dysfunction (34). The primary mechanism involves a decline in pumping capacity, reducing effective circulatory volume (35). Notably, excessive blood pressure reduction may compromise organ perfusion, potentially exacerbating renal impairment and increasing mortality risk. Valvular disorders or cardiac tamponade can further impair ventricular function and reduce coronary perfusion. As a compensatory response, sympathetic activation induces peripheral vasoconstriction to maintain coronary perfusion pressure, albeit at the cost of increased cardiac afterload (36). Consequently, managing hypotension in HF requires a delicate balance between maintaining vital organ perfusion and avoiding increased cardiac workload. Therefore, blood pressure serves as a significant indicator for risk prediction and assessment within the ERAS framework.

### 4.4 Limitations

Several limitations should be considered. First, the non-randomized design and baseline differences introduce potential for confounding. Second, the lack of objective muscle mass measurements limits direct assessment of sarcopenia. Third, the findings are limited to the perioperative period, and long-term functional outcomes were not assessed. While we infer SGLT2i's sarcopenic effects from functional scores, future studies should incorporate these direct measurements to quantitatively validate the drug's impact on skeletal muscle and its interaction with rehabilitation. Future multicenter studies should validate these findings, develop individualized rehabilitation strategies for VHD patients, and enhance perioperative monitoring of limb circumference.

## 5 Conclusion

In conclusion, SGLT2i use was associated with transient early mobility impairment, evidenced by delayed ICU discharge and first off-bed mobilization. However, within a structured ERAS protocol, these patients achieved superior functional recovery by the time of hospital discharge. This highlights the critical role of ERAS, particularly its early mobilization component, in mitigating initial deficits and facilitating recovery in patients on SGLT2 inhibitors.

## Data Availability

The original contributions presented in the study are included in the article/Supplementary Material, further inquiries can be directed to the corresponding authors.
