# Supplementary material for: Enhanced recovery mitigates sodium-glucose cotransporter-2 inhibitors associated mobility decline in valve surgery patients
Source: Front Cardiovasc Med. 2026 Feb 24;13:1746050. doi: 10.3389/fcvm.2026.1746050 (PMC12971437; doi:10.3389/fcvm.2026.1746050)
Supplement: Supplementary file 1 [file Table1.docx]

Supplementary Material

Table S1. Demographic characteristics and Surgery type of patients.

|  |  | QT(n=34) | TT(n=14) | P value | Z value a/χ2 b |
| --- | --- | --- | --- | --- | --- |
| Demographic characteristics | Age(years) | 53.50(45.50,61.50) | 54.00(45.80,66.00) | 0.81 | -0.238 |
|  | Sex(female/male) | 14/20 | 8/6 | 0.87 | -0.027 |
|  | DBP (mmHg) | 165.50(156.80,174.30) ** | 189.50(185.80,193.30) | ＜0.01 | -5.398 |
|  | SBP (mmHg) | 92.50(84.80,101.30) ** | 116.50(112.80,120.30) | ＜0.01 | -5.399 |
|  | Heart Rate(B/min) | 78.00(67.50,88.80) | 75.50(68.30,93.00) | 0.964 | -0.045 |
|  | NYHA functional class I | 8 | 4 |  |  |
|  | NYHA functional class II-III | 26 | 10 |  |  |
| Surgery type | Mitral valve | 6 | 1 |  |  |
|  | Tricuspid valve | 6 | 1 |  |  |
|  | Aortic valve | 3 | 4 |  |  |
|  | Mitral valve + tricuspid valve | 11 | 4 |  |  |
|  | Aortic valve+ Mitral valve | 2 | 3 |  |  |
|  | Trible valves | 6 | 1 |  |  |

^a^ Non-parametric analyses were performed using the Mann-Whitney U test with Bonferroni correction for multiple comparison; b chi-square test **p<0.01

Table S2 Functional mobility evaluation scale

| ​Scale​ | ​Indicator​ | ​Detailed Rules​ | ​Score​ |
| --- | --- | --- | --- |
| Barthel Index | Feeding | Independent | 10 |
|  |  | Needs help | 5 |
|  |  | Unable | 0 |
|  | Bathing | Independent | 5 |
|  |  | Unable | 0 |
|  | Grooming | Independent | 5 |
|  |  | Unable | 0 |
|  | Bowel control | Continent | 10 |
|  |  | Occasional accident | 5 |
|  |  | Incontinent (or needs to be given enemas) | 0 |
|  | Bladder control | Continent | 10 |
|  |  | Occasional accident | 5 |
|  |  | Incontinent (or needs to be given enemas) | 0 |
|  | Dressing | Independent | 10 |
|  |  | Needs help | 5 |
|  |  | Unable | 0 |
|  | Toilet use | Independent | 10 |
|  |  | Needs help | 5 |
|  |  | Unable | 0 |
|  | Transfers (bed to chair and back) | Independent | 15 |
|  |  | Needs minor help (verbal or physical) | 10 |
|  |  | Needs major help (1-2 people, physical), can sit | 5 |
|  |  | Unable | 0 |
|  | Mobility on level surfaces | Independent (but may use any aid,e.g. stick)>50 yards | 15 |
|  |  | Walks with help of one person (verbal or physical)>50 yards | 10 |
|  |  | Wheelchair independent, including corners,>50 yards | 5 |
|  |  | Immobile or<50 yards | 0 |
|  | Stairs | Independent | 10 |
|  |  | Needs help (verbal, physical, carrying aid) | 5 |
|  |  | Unable | 0 |
| Braden Scale | Sensory perception | Completely Limited | 1 |
|  |  | Very Limited | 2 |
|  |  | Slightly Limited | 3 |
|  |  | No Impairment | 4 |
|  | Moisture | Constantly Moist | 1 |
|  |  | Very Moist | 2 |
|  |  | Occasionally Moist | 3 |
|  |  | Rarely Moist | 4 |
|  | Activity | Bedfast | 1 |
|  |  | Chairfast | 2 |
|  |  | Walks Occasionally | 3 |
|  |  | Walks Frequently | 4 |
|  | Mobility | Completely Immobile | 1 |
|  |  | Very Limited | 2 |
|  |  | Slightly Limited | 3 |
|  |  | No Limitation | 4 |
|  | Nutrition | Very Poor | 1 |
|  |  | Probably Inadequate | 2 |
|  |  | Adequate | 3 |
|  |  | Excellent | 4 |
|  | Friction & shear | Problem | 1 |
|  |  | Potential Problem | 2 |
|  |  | No Apparent Problem | 3 |
| Thomas Fall Risk Scale | History of falling | yes | 1 |
|  |  | no | 0 |
|  | Cognitive impairment | yes | 1 |
|  |  | no | 0 |

Table S1 Adapted from Wang et al. [9-11]

Table S3. Baseline characters of patients.

|  |  | QT(n=34) | TT(n=14) | P value a | Z value |
| --- | --- | --- | --- | --- | --- |
| Cardiac function | Ejection fraction (%) | 66.00(58.00,71.25) | 64.00(61.00,69.25) | 0.867 | -0.167 |
|  | Fractional shortening (%) | 37.00(30.00,41.00) | 33.50(32.00,38.00) | 0.596 | -0.530 |
|  | Left atrium(mm) | 53.00(44.75,56.25) | 51.00(45.50,53.00) | 0.387 | -0.866 |
|  | Aorta ascendens(mm) | 35.00(32.00,40.25) | 34.00(29.50,40.00) | 0.564 | -0.577 |
|  | Right atrium(mm) | 26.00(23.75,29.25) | 26.00(23.50,27.50) | 0.792 | -0.264 |
|  | Main pulmonary artery(mm) | 28.50(26.00,33.00) | 26.00(24.50,30.50) | 0.096 | -1.667 |
|  | Interventricular septum(mm) | 10.40(9.43,12.52) | 11.40(9.65,13.25) | 0.341 | -0.953 |
|  | Left ventricular end diastolic dimension (mm) | 53.00(46.50,61.25) | 54.00(46.00,58.00) | 0.625 | -0.489 |
|  | left ventricular end-systolic dimension(mm) | 33.00(30.75,38.00) | 35.00(30.50,45.00) | 0.831 | -0.213 |
|  | Left ventricular posterior wall dimension (mm) | 9.95(8.93,11.70) | 10.20(8.55,11.85) | 0.867 | -0.167 |
| Mobility | Activities of Daily Living scale | 100.00(95.00,100.00) | 100.00(100.00,100.00) | 0.294 | -1.049 |
|  | Braden scale | 23.00(22.00，23.00) | 23.00(23.00，23.00) | 0.117 | -1.567 |
|  | Modified Thomas fall risk assessment scale | 1.00(0.75，2.00) | 1.00(0.00，2.00) | 0.423 | -0.801 |
| Laboratory test | Red blood cell(109/l) | 4.40(3.81, 4.93) | 4.68(4.26,4.89) | 0.335 | -0.963 |
|  | Hemoglobin(g/l) | 131.00(108.00, 146.00) | 140.00(129.50,152.50) | 0.267 | -1.111 |
|  | White blood cell(109/l) | 7.23(5.29, 8.71) | 5.72(4.75,8.69) | 0.285 | -1.07 |
|  | Platelet(109/l) | 244.00(186.75, 275.50 | 206.00(149.00,230.50) | 0.069 | -1.819 |
|  | Epidermal growth factor receptor(mL/min/1.73m2) | 96.40(77.85, 105.65) | 97.20(75.5,108.35) | 0.77 | -0.293 |
|  | Red blood cell(109/l) | 4.40(3.81, 4.93) | 4.68(4.26,4.89) | 0.335 | -0.963 |
|  | Hemoglobin(g/l) | 131.00(108.00, 146.00) | 140.00(129.50,152.50) | 0.267 | -1.111 |
|  | White blood cell(109/l) | 7.23(5.29, 8.71) | 5.72(4.75,8.69) | 0.285 | -1.07 |

^a^ Non-parametric analyses were performed using the Mann-Whitney U test with Bonferroni correction for multiple comparison.

Table S4. Intra- and Inter-group Comparison of functional mobility assessment

| Scale | Time point | QT(n=34) | TT(n=14) | p value Inter-group ^a^ | p value Intra-group ^b^ | |
| --- | --- | --- | --- | --- | --- | --- |
|  |  |  |  |  | QT | TT |
| Activities of Daily Living scale​ | Admission | 100.00(95.00,100.00) | 100.00(100.00,100.00) | 0.294 | 9.3899E-33 | 4.7208E-12 |
|  | Immediately post-surgery | 0.00(0.00,10.00) ** | 2.50(0.00,10.00) | 0.097 |  |  |
|  | Awakening from anesthesia | 10.00(10.00,10.00) ** | 15.00(10.00,22.50) | 0.007 |  |  |
|  | Discharge from ICU | 30.00(20.00,35.00) | 25.00(20.00,30.00) | 0.200 |  |  |
|  | Secondary care phase | 95.00(85.00,100.00) * | 85.00(70.00,90.00) | 0.017 |  |  |
|  | Discharge | 100.00(98.75,100.00) | 100.00(92.00,100.00) | 0.445 |  |  |
| Braden scale​ | Admission | 23.00(22.00,23.00) | 23.00(23.00,23.00) | 0.117 | 2.4036E-32 | 2.1545E-11 |
|  | Immediately post-surgery | 10.50(10.00,13.00) | 12.50(10.00,15.00) | 0.282 |  |  |
|  | Awakening from anesthesia | 13.00(12.00,15.00) * | 16.00(14.50,17.50) | 0.025 |  |  |
|  | Discharge from ICU | 16.00(15.00,17.00) ** | 17.00(16.00,18.00) | 0.006 |  |  |
|  | Secondary care phase | 22.00(20.75,23.00) * | 21.00(20.00,21.00) | 0.043 |  |  |
|  | Discharge | 23.00(22.00,23.00) | 23.00(22.00,23.00) | 0.088 |  |  |
| Modified Thomas fall risk assessment scale​ | Admission | 1.00(0.00,2.00) | 1.00(0.00,2.00) | 0.423 | 0.014 | 0.014 |
|  | Immediately post-surgery | 2.00(1.00,4.00) | 2.00(1.00,2.00) | 0.122 |  |  |
|  | Awakening from anesthesia | 2.00(1.00,4.00) | 1.00(1.00,2.00) | 0.331 |  |  |
|  | Discharge from ICU | 1.50(1.00,2.00) | 1.50(1.00,2.00) | 0.411 |  |  |
|  | Secondary care phase | 1.00(1.00,3.00) | 2.00(1.00,4.00) | 0.445 |  |  |
|  | Discharge | 1.00(1.00,2.00) | 1.00(1.00,2.00) | 0.474 |  |  |

a Non-parametric analyses were performed using the Mann-Whitney U test with Bonferroni correction for multiple comparison, * p<0.05, **p<0.01. b Friedman test, * p<0.05, **p<0.01

Table S5. Comparison of postoperative Cardiac function and Laboratory test.

|  |  |  | QT(n=34) | TT(n=14) |  |  |
| --- | --- | --- | --- | --- | --- | --- |
|  |  |  | M (Q1, Q3) | M (Q1, Q3) | Z value | p Value ^a^ |
| Cardiac function | Ejection fraction (%) | Postoperative | 64.00(56.80,66.00) | 53.00(43.00,65.00) | -1.899 | 0.058 |
|  |  | Discharge | 59.00(47.00,66.00) | 61.00(53.50,65.00) | -0.023 | 0.982 |
|  | Fractional shortening (%) | Postoperative | 44.00(38.00,52.00) | 45.00(39.50,52.50) | -0.251 | 0.802 |
|  |  | Discharge | 46.00(41.00,50.00) | 46.00(38.00,53.00) | -0.149 | 0.881 |
|  | Left atrium(mm) | Postoperative | 28.00(25.00,34.00) | 32.00(26.00,36.80) | -0.829 | 0.407 |
|  |  | Discharge | 27.00(24.50,32.50) | 32.00(25.50,35.50) | -1.313 | 0.189 |
|  | Aorta ascendens(mm) | Postoperative | 24.00(22.00,29.00) | 27.00(22.00,32.0) | -0.689 | 0.491 |
|  |  | Discharge | 30.50(19.50,37.00) | 25.50(22.00,27.50) | -0.896 | 0.37 |
|  | Right atrium(mm) | Postoperative | 27.00(24.00,30.00) | 29.00(26.50,30.50) | -0.895 | 0.371 |
|  |  | Discharge | 28.00(26.00,33.00) | 26.50(23.80,32.00) | -0.818 | 0.413 |
|  | Main pulmonary artery(mm) | Postoperative | 11.00(9.90,13.80) | 10.30(9.20,13.70) | -0.609 | 0.542 |
|  |  | Discharge | 10.20 (9.30,14.00) | 10.60(9.70,12.60) | -0.213 | 0.831 |
|  | Interventricular septum(mm) | Postoperative | 49.00(44.30,50.80) | 46.00(43.00,56.50) | -0.105 | 0.916 |
|  |  | Discharge | 47.00(42.00,50.00) | 49.00(43.50,56.00) | -0.883 | 0.377 |
|  | Left ventricular end diastolic dimension (mm) | Postoperative | 32.00(30.30,33.00) | 34.00(29.50,46.00) | -1.116 | 0.264 |
|  |  | Discharge | 30.00(24.00,36.00) | 32.50(28.00,40.50) | -1.125 | 0.261 |
|  | left ventricular end-systolic dimension(mm) | Postoperative | 44.00(38.00,52.00) | 45.00(39.50,52.50) | -0.251 | 0.802 |
|  |  | Discharge | 46.00(41.00,50.00) | 46.00(38.00,53.00) | -0.149 | 0.881 |
|  | Left ventricular posterior wall dimension (mm) | Postoperative | 28.00(25.00,34.0)0 | 32.00(26.00,36.80) | -0.829 | 0.407 |
|  |  | Discharge | 27.00(24.50,32.50) | 32.00(25.50,35.50) | -1.313 | 0.189 |
| Laboratory test | White blood cell(109/l) | Postoperative | 16.88(12.40,25.10) | 17.85 (12.90,20.40) | -0.091 | 0.928 |
|  |  | Discharge | 9.97(7.20,12.20) | 8.96 (7.60,10.40) | -0.547 | 0.585 |
|  | Red blood cell(109/l) | Postoperative | 4.09(3.80,4.30) | 3.84(3.50,4.40) | -0.805 | 0.421 |
|  |  | Discharge | 3.54 (3.10,3.80) | 3.37 (3.00,3.80) | -0.512 | 0.609 |
|  | Hemoglobin(g/l) | Postoperative | 118.00(106.80,127.30) | 117.50 (95.80,135.00) | -0.102 | 0.919 |
|  |  | Discharge | 101.00(90.80,110.30) | 96.00 (89.00,112.00) | -0.221 | 0.825 |
|  | Platelet(109/l) | Postoperative | 110.00(78.00,167.80) | 145.00(112.30,183.00) | -1.497 | 0.134 |
|  |  | Discharge | 307.50(233.80,424.50) | 322.00(203.50,375.00) | -0.465 | 0.642 |
|  | Epidermal growth factor(mL/min/1.73m2) | Postoperative | 90.20(61.00,109.90) | 90.50(61.90,103.20) | -0.031 | 0.976 |
|  |  | Discharge | 105.70(84.30,109.10) | 102.25(72.30,111.20) | -0.3 | 0.764 |
|  | High-sensitivity C-reactive protein(mg/l) | Postoperative | 87.72(19.90,111.00) | 58.44(30.40,82.90) | -0.844 | 0.399 |
|  |  | Discharge | 44.68(28.80,78.50) | 45.770(23.80,73.60) | -0.059 | 0.953 |
|  | Albumin(g/dL) | Postoperative | 35.55(31.00,37.00) | 35.70(33.40,38.00) | -0.963 | 0.336 |
|  |  | Discharge | 36.35(34.30,37.80) | 36.40(35.10,39.30) | -0.443 | 0.658 |
|  | NT-pro BNP | Postoperative | 655.50(395.30,1194.80) | 416.00(249.00,1347.50) | -1.023 | 0.306 |
|  |  | Discharge | 825.00(370.30,1284.30) | 480.00(280.10,1076.60) | -1.002 | 0.317 |

^a^ Non-parametric analyses were performed using the Mann-Whitney U test with Bonferroni correction for multiple comparison

Table S6. Correlation of ERAS report elements and Mobility assessment

| Duration of operation | Duration of ICU | Duration of mechanical ventilation | Time of first in-bed mobilization post- surgery | Time of first off-bed mobilization post- surgery | Postoperative hospitalization |
| --- | --- | --- | --- | --- | --- |
| ADL Hospitalization | -0.072 | -0.263 | -0.043 | 0.166 | -0.073 |
| ADL Immediately post- surgery | 0.181 | -0.02 | -0.035 | 0.174 | -0.085 |
| ADL Awake under general anesthesia | -0.076 | -0.208 | -0.243 | 0.191 | -0.049 |
| ADL Discharge from ICU | 0.012 | 0.482** | 0.568** | 0.333* | 0.261 |
| ADL Secondary care phase | -0.056 | 0.042 | 0.113 | 0.119 | -0.056 |
| ADL Discharge | -0.196 | -0.296* | 0.004 | 0.005 | -0.094 |
| Braden Hospitalization | 0.039 | -0.249 | 0.039 | 0.181 | -0.161 |
| Braden Immediately post- surgery | 0.023 | -0.156 | -0.087 | 0.211 | -0.023 |
| Braden Awake under general anesthesia | 0.029 | -0.221 | -0.350* | 0.07 | -0.086 |
| Braden Discharge from ICU | 0.008 | -0.06 | -0.09 | 0.153 | -0.017 |
| Braden Secondary care phase | -0.011 | 0.059 | 0.174 | 0.202 | 0.141 |
| Braden Discharge | -0.066 | 0.043 | 0.073 | 0.155 | -0.033 |
| TRAFS Hospitalization | 0.218 | 0.213 | 0.119 | -0.126 | 0.055 |
| TRAFS Immediately post- surgery | 0.302* | -0.142 | -0.155 | -0.084 | -0.194 |
| TRAFS Awake under general anesthesia | 0.101 | -0.083 | -0.137 | -0.071 | -0.108 |
| TRAFS Discharge from ICU | 0.241 | -0.005 | -0.086 | -0.129 | -0.075 |
| TRAFS Secondary care phase | 0.128 | 0.133 | -0.067 | -0.201 | -0.054 |
| TRAFS Discharge | 0.012 | 0.136 | -0.056 | -0.262 | 0.07 |

Spearman Correlation test, * p<0.05, **p<0.01

Table S7. Correlation of ERAS report elements and Mobility assessment

|  | ADL Hospitalization | ADL Immediately post- surgery | ADL Awake under general anesthesia | ADL Discharge from ICU | ADL Secondary care phase | ADL Discharge | Braden Hospitalization | Braden Immediately post- surgery | Braden Awake under general anesthesia | Braden Discharge from ICU | Braden Secondary care phase | Braden Discharge | TRAFS scHospitalization | TRAFS Immediately post- surgery | TRAFS Awake under general anesthesia | TRAFS Discharge from ICU | TRAFS Secondary care phase | TRAFS Discharge |
| --- | --- | --- | --- | --- | --- | --- | --- | --- | --- | --- | --- | --- | --- | --- | --- | --- | --- | --- |
| WBC Hospitalization | -0.11 | 0.164 | -0.129 | 0.042 | -0.239 | -0.101 | -0.119 | 0.221 | 0.118 | 0.032 | -0.222 | -0.203 | 0.093 | 0.05 | 0.1 | -0.21 | 0.159 | -0.079 |
| WBC postoperative1 | 0.095 | -0.046 | -0.146 | 0.206 | -0.083 | 0.072 | 0.084 | 0.099 | 0.201 | 0.173 | -0.033 | -0.129 | -0.13 | 0.023 | 0.077 | -0.084 | -0.029 | -0.206 |
| WBC Discharge | 0.202 | 0.009 | -0.027 | 0.249 | 0.05 | 0.052 | 0.245 | 0.048 | 0.017 | -0.06 | 0.025 | 0.092 | -0.276 | 0.006 | 0.065 | -0.039 | -0.112 | -0.19 |
| RBC Hospitalization | 0.199 | -0.358* | 0 | -0.048 | 0.036 | 0.187 | 0.255 | 0.153 | 0.143 | -0.167 | 0.108 | 0.185 | -0.243 | -0.104 | -0.036 | -0.053 | -0.007 | -0.156 |
| RBC postoperative1 | 0.212 | -0.239 | -0.033 | -0.045 | 0.191 | 0.155 | 0.195 | 0.146 | 0.216 | -0.02 | 0.174 | 0.078 | -0.329 | -0.207 | -0.03 | 0.152 | -0.05 | -0.119 |
| RBC Discharge | 0.168 | -0.231 | -0.062 | -0.015 | 0.072 | 0.068 | -0.038 | 0.196 | 0.097 | 0.16 | 0.291 | 0.176 | -0.278 | -0.18 | 0.121 | 0.224 | -0.017 | 0.035 |
| PLT Hospitalization | 0.07 | 0.01 | 0 | 0.203 | 0.086 | 0.016 | -0.254 | -0.102 | -0.055 | 0.317 | 0.109 | 0.034 | -0.069 | -0.085 | -0.044 | -0.25 | -0.133 | -0.178 |
| PLT postoperative1 | 0.184 | -0.135 | -0.055 | 0.268 | 0.217 | 0.279 | -0.17 | -0.038 | 0.044 | 0.342* | 0.167 | 0.085 | -0.217 | -0.153 | -0.139 | -0.346* | -0.191 | -0.382* |
| PLT Discharge | 0.141 | 0.034 | 0.135 | 0.294 | 0.347* | 0.208 | -0.187 | 0.125 | 0.103 | 0.367* | 0.185 | 0.211 | -0.149 | -0.332 | -0.231 | -0.417* | -0.158 | -0.495** |
| Hgb Hospitalization | 0.07 | 0.01 | 0 | 0.203 | 0.086 | 0.016 | -0.254 | -0.102 | -0.055 | 0.317 | 0.109 | 0.034 | -0.069 | -0.085 | -0.044 | -0.25 | -0.133 | -0.178 |
| Hgb postoperative1 | 0.184 | -0.135 | -0.055 | 0.268 | 0.217 | 0.279 | -0.17 | -0.038 | 0.044 | 0.342* | 0.167 | 0.085 | -0.217 | -0.153 | -0.139 | -0.346* | -0.191 | -0.382* |
| Hgb Discharge | 0.141 | 0.034 | 0.135 | 0.294 | 0.347* | 0.208 | -0.187 | 0.125 | 0.103 | 0.367* | 0.185 | 0.211 | -0.149 | -0.332 | -0.231 | -0.417* | -0.158 | -0.495** |
| eGFR preoperative | 0.055 | -0.226 | -0.213 | -0.366* | 0.002 | 0.001 | 0.019 | -0.163 | 0.238 | -0.122 | 0.177 | 0.058 | -0.24 | 0.14 | -0.07 | 0.045 | -0.265 | 0.01 |
| eGFR postoperative | 0.056 | -0.029 | 0.038 | -0.445** | 0.271 | -0.064 | -0.087 | 0.11 | 0.487** | -0.159 | 0.203 | -0.062 | -0.317 | -0.005 | 0.036 | 0.045 | -0.251 | 0.05 |
| eGFR Discharge | -0.112 | -0.028 | -0.12 | -0.293 | -0.177 | -0.141 | 0.002 | -0.082 | 0.105 | -0.126 | -0.074 | -0.18 | -0.271 | 0.049 | -0.194 | -0.054 | -0.224 | 0.026 |
| hs-CRP preoperative | -0.288 | -0.031 | -0.028 | 0.179 | -0.072 | 0.036 | -0.390* | 0.138 | 0.302 | 0.097 | -0.125 | -0.003 | -0.17 | 0.072 | -0.252 | -0.317 | 0.031 | -0.292 |
| hs-CRP postoperative | -0.051 | -0.047 | 0.446* | 0.202 | 0.159 | 0.192 | -0.156 | 0.203 | 0.352 | -0.088 | 0.065 | 0.347 | -0.148 | 0.098 | 0.119 | -0.104 | 0.163 | -0.175 |
| hs-CRP Discharge | 0.003 | -0.117 | -0.046 | 0.184 | -0.107 | -0.177 | 0.089 | 0.118 | -0.124 | -0.072 | 0.084 | -0.021 | -0.237 | -0.332 | 0.117 | 0.169 | -0.153 | 0.138 |
| Albumin preoperative | 0.214 | 0.166 | 0.031 | -0.096 | 0.294 | 0.188 | 0.297 | -0.11 | -0.013 | -0.470** | 0.098 | 0.298 | -0.038 | 0.039 | 0.087 | 0.068 | 0.18 | 0.01 |
| Albumin postoperative | 0.254 | -0.111 | -0.131 | -0.117 | 0.387* | 0.369* | 0.034 | 0.13 | 0.288 | 0.156 | 0.263 | 0.155 | -0.203 | -0.076 | -0.005 | -0.213 | -0.287 | -0.345* |
| Albumin discharge | 0.194 | 0.139 | 0.2 | 0.198 | 0.323 | 0.198 | -0.031 | 0.122 | 0.262 | 0.178 | 0.127 | -0.022 | -0.288 | -0.291 | -0.147 | -0.183 | 0.03 | -0.364* |
| NTproBNP admission | -0.225 | 0.398* | 0.181 | 0.1 | -0.31 | -0.246 | -0.135 | 0.073 | -0.367* | 0.212 | -0.271 | -0.318 | 0.470** | 0.193 | -0.068 | -0.06 | 0.165 | -0.072 |
| NTproBNP postoperative | -0.026 | 0.214 | 0.214 | -0.201 | -0.259 | -0.128 | -0.241 | 0.166 | -0.101 | 0.149 | -0.267 | -0.269 | 0.438** | 0.15 | 0.018 | 0.088 | 0.141 | 0.016 |
| NTproBNP discharge | 0.085 | 0.184 | -0.018 | 0.198 | -0.480** | -0.360* | 0.068 | -0.227 | -0.585** | 0.267 | -0.283 | -0.152 | 0.283 | -0.066 | 0.167 | 0.214 | 0.079 | 0.201 |

Spearman Correlation test, * p<0.05, **p<0.01

Table S8. Correlation of Cardiac function Mobility assessment

|  | ADL Hospitalization | ADL Immediately post- surgery | ADL Awake under general anesthesia | ADL Discharge from ICU | ADL Secondary care phase | ADL Discharge | Braden Hospitalization | Braden Immediately post- surgery | Braden Awake under general anesthesia | Braden Discharge from ICU | Braden Secondary care phase | Braden Discharge | TRAFS scHospitalization | TRAFS Immediately post- surgery | TRAFS Awake under general anesthesia | TRAFS Discharge from ICU | TRAFS Secondary care phase | TRAFS Discharge |
| --- | --- | --- | --- | --- | --- | --- | --- | --- | --- | --- | --- | --- | --- | --- | --- | --- | --- | --- |
| LA preoperative | 0.048 | -0.013 | 0.048 | 0.216 | 0.083 | 0.043 | -0.053 | -0.093 | -0.091 | 0.184 | 0.039 | 0.202 | -0.189 | -0.176 | -0.052 | -0.294 | -0.112 | -0.186 |
| LA postoperative | 0.007 | -0.199 | -0.252 | -0.022 | 0.077 | -0.038 | -0.011 | -0.119 | -0.122 | 0.112 | 0.006 | 0.231 | -0.072 | -0.043 | -0.241 | -0.035 | -0.147 | -0.056 |
| LA Discharge | 0.032 | -0.042 | 0.051 | 0.088 | 0.09 | -0.013 | -0.128 | 0.027 | -0.053 | 0.105 | -0.066 | 0.206 | -0.087 | -0.151 | -0.197 | -0.375 | -0.199 | -0.217 |
| AAO preoperative | -0.123 | -0.166 | 0.125 | -0.127 | -0.268 | -0.153 | -0.21 | 0.151 | -0.089 | 0.277 | 0.162 | 0.167 | 0.271 | 0.113 | 0.08 | 0.255 | 0.062 | 0.392* |
| AAO postoperative | 0.182 | 0.032 | -0.016 | -0.124 | 0.420* | 0.082 | 0.06 | 0.098 | -0.168 | 0.382 | 0.438* | 0.173 | 0.254 | -0.017 | -0.321 | 0.081 | -0.298 | -0.143 |
| AAO Discharge | -0.316 | -0.022 | 0.302 | -0.05 | 0.149 | 0.059 | -0.416* | 0.264 | 0.029 | 0.153 | 0.283 | -0.206 | 0.481* | 0.27 | -0.119 | 0.215 | 0.011 | 0.171 |
| RA preoperative | -0.093 | 0.03 | -0.303 | -0.096 | 0.155 | 0.059 | -0.121 | -0.069 | -0.318 | 0.049 | 0.185 | 0.238 | 0.034 | -0.31 | -0.098 | 0.125 | 0.019 | 0.046 |
| RA postoperative | -0.313 | 0.071 | -0.044 | -0.085 | -0.364 | 0.202 | -0.323 | 0.382 | -0.136 | -0.098 | -0.128 | 0.274 | -0.031 | -0.254 | -0.081 | -0.157 | 0.185 | 0.096 |
| RA Discharge | 0.053 | 0.112 | -0.259 | 0.208 | -0.29 | -0.115 | -0.149 | 0.109 | -0.247 | 0.328 | 0.068 | 0.325 | -0.066 | -0.432* | 0.23 | -0.034 | 0.267 | 0.081 |
| MPA preoperative | -0.079 | 0.173 | -0.177 | -0.182 | -0.008 | -0.165 | -0.095 | 0.103 | -0.196 | 0.388* | 0.265 | 0.053 | 0.118 | -0.22 | -0.127 | 0.231 | -0.055 | 0.195 |
| MPA postoperative | -0.096 | 0.157 | 0.418 | 0.106 | 0.314 | -0.1 | -0.283 | 0.036 | -0.157 | 0.124 | 0.256 | 0.451 | -0.199 | -0.218 | 0.02 | -0.059 | -0.1 | -0.134 |
| MPA Discharge | 0.103 | 0.139 | 0.083 | -0.081 | 0.262 | 0.034 | -0.12 | 0.202 | 0.192 | 0.302 | 0.298 | 0.244 | -0.236 | -0.304 | -0.018 | -0.131 | -0.383 | -0.015 |
| IVS preoperative | -0.006 | -0.198 | -0.088 | -0.018 | -0.119 | 0.111 | -0.237 | 0.257 | -0.065 | 0.087 | 0.112 | 0.482** | -0.059 | -0.221 | -0.038 | -0.064 | 0.17 | -0.031 |
| IVS postoperative | -0.209 | -0.216 | 0.027 | -0.093 | -0.166 | 0.119 | -0.446* | 0.197 | 0.114 | 0.093 | 0.138 | 0.406* | 0.018 | 0.051 | 0.148 | -0.012 | 0.111 | 0.082 |
| IVS Discharge | 0.028 | -0.016 | -0.165 | -0.03 | -0.111 | -0.257 | 0.075 | 0.11 | 0.089 | 0.223 | 0.305 | 0.296 | 0.143 | 0.193 | 0.28 | 0.308 | 0.023 | 0.244 |
| LVD preoperative | -0.403* | 0.208 | 0.152 | 0.154 | -0.113 | -0.439* | -0.494** | 0.202 | 0.102 | 0.265 | -0.088 | -0.14 | -0.036 | -0.091 | -0.041 | -0.037 | 0.003 | -0.001 |
| LVD postoperative | -0.313 | 0.036 | 0.009 | 0.162 | 0.053 | -0.351 | -0.31 | 0.138 | -0.071 | 0.155 | -0.075 | 0.095 | -0.114 | -0.281 | -0.186 | -0.176 | 0.042 | -0.303 |
| LVD Discharge | -0.16 | -0.067 | -0.118 | 0.379 | 0.008 | -0.548** | -0.419* | 0.175 | -0.186 | 0.249 | 0.001 | 0.164 | -0.205 | -0.426* | 0.053 | -0.102 | 0.012 | -0.156 |
| LVS preoperative | -0.467** | 0.211 | 0.014 | 0.189 | -0.244 | -0.543** | -0.382* | 0.18 | -0.045 | 0.321 | -0.118 | -0.208 | 0.139 | 0.055 | 0.034 | 0.029 | -0.008 | 0.18 |
| LVS postoperative | -0.309 | -0.244 | -0.185 | 0.037 | 0.167 | -0.283 | -0.317 | -0.085 | -0.283 | 0.243 | 0.041 | 0.118 | -0.006 | -0.294 | -0.201 | -0.099 | 0.012 | -0.219 |
| LVS Discharge | -0.235 | -0.19 | -0.081 | 0.291 | -0.02 | -0.448* | -0.395* | 0.096 | -0.164 | 0.246 | -0.066 | 0.133 | -0.183 | -0.371 | -0.015 | -0.173 | -0.033 | -0.141 |
| LPWD preoperative | 0.054 | -0.213 | -0.286 | -0.225 | -0.191 | 0.019 | -0.096 | 0.168 | -0.259 | 0.176 | 0.147 | 0.398* | 0.026 | -0.229 | 0.094 | 0.052 | 0.024 | 0.118 |
| LPWD postoperative | -0.038 | -0.077 | 0.002 | 0.041 | -0.205 | 0.077 | -0.283 | -0.1 | -0.104 | 0.299 | 0.041 | 0.256 | -0.014 | 0.012 | 0.026 | -0.088 | 0.033 | 0.012 |
| LPWD Discharge | 0.091 | -0.113 | -0.171 | 0.467* | -0.161 | -0.187 | -0.172 | -0.069 | -0.284 | 0.541** | 0.242 | 0.379 | -0.065 | -0.095 | 0.185 | 0.235 | -0.214 | 0.113 |
| EF preoperative | 0.218 | -0.114 | 0.097 | -0.054 | 0.206 | 0.128 | -0.075 | -0.044 | 0.304 | 0.029 | 0.144 | 0.038 | -0.119 | -0.035 | 0 | 0.044 | 0.054 | -0.049 |
| EF postoperative | 0.156 | 0.305 | 0.099 | -0.4 | -0.112 | 0.453* | 0.203 | 0.323 | 0.273 | -0.345 | 0.092 | 0.119 | 0.023 | 0.098 | 0.022 | 0.2 | 0.198 | 0.344 |
| EF Discharge | 0.342 | 0.321 | -0.011 | -0.149 | -0.063 | 0.183 | 0.492* | -0.033 | 0.108 | -0.311 | -0.057 | -0.067 | -0.053 | 0.197 | 0.064 | 0.169 | -0.034 | -0.062 |
| FS preoperative | 0.22 | -0.181 | -0.163 | -0.18 | 0.282 | 0.164 | -0.056 | -0.097 | 0.108 | 0.045 | 0.234 | 0.106 | -0.057 | -0.192 | -0.087 | 0.162 | 0.061 | -0.042 |
| FS postoperative | 0.168 | 0.525* | 0.202 | -0.288 | -0.056 | 0.491* | 0.278 | 0.326 | 0.488* | -0.326 | 0.074 | 0.02 | -0.011 | 0.228 | 0.077 | 0.127 | 0.084 | 0.287 |
| FS Discharge | 0.361 | 0.316 | -0.039 | -0.132 | -0.12 | 0.107 | 0.488* | -0.032 | 0.125 | -0.308 | -0.191 | -0.123 | -0.126 | 0.147 | -0.048 | 0.1 | -0.109 | -0.187 |

Spearman Correlation test, * p<0.05, **p<0.01
