# Supplementary material for: Enhanced recovery mitigates sodium-glucose cotransporter-2 inhibitors associated mobility decline in valve surgery patients
Source: Front Cardiovasc Med. 2026 Feb 24;13:1746050. doi: 10.3389/fcvm.2026.1746050 (PMC12971437; doi:10.3389/fcvm.2026.1746050)
Supplement: Supplementary file 2 [file Datasheet1.pdf]

厦门大学附属中山医院
ZHONGSHAN HOSPITAL XIAMEN UNIVERSITY

医学伦理委员会科研分委会伦理审查批件

批件号：xmzsyyky伦审第（2024-113）

审查时间	2024 年 8 月 1 日
项目名称	基于多组学方法的心脏大血管加速康复外科（ERAS-C）评定生物标志物探索研究
项目负责人	孙勇
项目来源	自筹
受理号	2024-113
审查文件	《医学伦理委员会科研分委会伦理审查申请表》<br>《项目负责人信息》<br>《研究方案》 版本号：V1.0 版本日期：2024年5月27日<br>《知情同意书》 版本号：V1.0 版本日期：2024年5月27日
审查方式	（标■表示） □会议审查 ■快速审查
审查类别	（标■表示） ■初始审查 □复 审
审查意见	结论：（标■表示） ■ 同意 □ 作必要修正后同意<br>□作必要修正后重审 □ 不同意
伦理委员会意见	本伦理委员会对该项目的研究方案、知情同意书等有关材料进行认真与充分的讨论，经审查项目符合伦理要求，同意开展研究。

主任或副主任委员签字：

[illegible] 2024 年 8 月 1 日

厦门大学附属中山医院医学伦理委员会（盖章）

注意：（请仔细阅读）

1、所有资料未经本伦理委员会批准，不得作任何修改，必须严格按照所批最新版本的研究方案和知情同意书开展研究；如有违背方案、更新申请材料、暂停/提前终止临床研究请及时通知伦理委员会；

2、凡是涉及人类遗传资源出口或者按照国家规定必须经有关部门专项审批的内容，均需在项目执行前向有关部门申报并获得批准；

3、发生严重不良事件及影响研究风险受益比的非预期事件，请24小时内报告本伦理委员会；

4、该项目自通过伦理审查之日起，必须半年内启动，若超过半年不启动，本批件自动作废；

5、该项目自通过伦理审查之日起，开展每满一年，均应向本伦理委员会提交年度研究进展报告；

6、研究结束时，请提交结题报告。

厦门大学附属中山医院医学伦理委员会科研分委会 联系电话：05922292476

地址：厦门市思明区湖滨南路201-209号 邮编：361004
